# Microfluidic Device with an Integrated Freeze-Dried Cell-Free Protein Synthesis System for Small-Volume Biosensing †

**DOI:** 10.3390/mi12010027

**Published:** 2020-12-29

**Authors:** Taishi Tonooka

**Affiliations:** Faculty of Mechanical Engineering, Kyoto Institute of Technology, Matsugasaki, Sakyo-ku, Kyoto 606-8585, Japan; tonooka@kit.ac.jp; Tel.: +81-75-724-7749

**Keywords:** freeze-dry, cell-free protein synthesis (CFPS), point-of-care (PoC), microfluidic device

## Abstract

Microfluidic devices enable the precise operation of liquid samples in small volumes. This motivates why microfluidic devices have been applied to point-of-care (PoC) liquid biopsy. Among PoC liquid biopsy studies, some report diagnostic reagents being freeze-dried in such microfluidic devices. This type of PoC microfluidic device has distinct advantages, such as simplicity of the procedures, compared with other PoC devices using liquid-type diagnostic reagents. Despite the attractive characteristic, only diagnostic reagents based on the cloned enzyme donor immunoassay (CEDIA) have been freeze-dried in the microfluidic device. However, development of the PoC device based on the CEDIA method is time-consuming and labor-intensive. Here, we employed a molecule-responsive protein synthesis system as the diagnostic reagent to be freeze-dried in the microfluidic device. Such molecule-responsive protein synthesis has been well investigated in the field of molecular biology. Therefore, using the accumulated information, PoC devices can be efficiently developed. Thus, we developed a microfluidic device with an integrated freeze-dried molecule-responsive protein synthesis system. Using the developed device, we detected two types of bio-functional molecules (i.e., bacterial quorum sensing molecules and mercury ions) by injecting 1 µL of sample solution containing these molecules. We showed that the developed device is applicable for small-volume biosensing.

## 1. Introduction

Microfluidic devices make it possible to analyze sample solutions even in small volumes. In fact, the sample volume required to analyze with microfluidic devices is smaller than that of a general plate reader (~5 µL). This advantage is useful to reduce invasiveness in liquid biopsy using blood, urine, saliva, and tears. Therefore, microfluidic devices have been applied as point-of-care (PoC) diagnostic devices [1,2,3,4]. Recently, an attractive PoC device has been developed [5] (i.e., a microfluidic device equipped with freeze-dried diagnostic reagents). Such device has a distinct advantage compared with other PoC devices using liquid-based diagnostic reagents: Simple operational procedure, users do not need to mix and/or apply diagnostic reagents when using the PoC device, and simple loading of the sample solution to the PoC device enables liquid biopsy. Although PoC devices with freeze-dried diagnostic reagents have these attractive characteristics, only the antigen-antibody diagnostic reagents based on the cloned enzyme donor immunoassay method have been freeze-dried so far. The cloned enzyme donor immunoassay (CEDIA) method requires an analyte–donor complex to test a specific analyte in a solution [6]. Preparing the analyte–donor complex is time-consuming and labor-intensive. Furthermore, it is uncertain whether the analyte–donor complex can be obtained. In addition, if the antigen–antibody detection principle is employed, except for the homogeneous immunoassay including the CEDIA method, complex procedures for immobilizing antibodies and/or washing unbound secondary antibodies are required [7]. Considering these aspects, a more developer-friendly and simple method based on a principle other than antigen–antibody diagnostic reagents is expected to broaden the use of PoC devices with freeze-dried diagnostic reagents.

The molecule-responsive protein expression system consists of a cell-free protein synthesis (CFPS) system and DNA encoding a reporter protein under a molecule-responsive promoter. CFPS systems generally contain RNA polymerase, ribosome, nucleotides, amino acids, adenosine triphosphate regeneration system, and salts [8,9]. The system produces signal proteins, such as the green fluorescent protein (GFP) when the target molecule triggers the molecule-responsive promoter, such that the user can monitor the concentration of the target molecule using fluoresce-based measurements [10]. Molecule-responsive promoters have been investigated for many years in the field of molecular biology. Therefore, there is plenty of information on various molecule-responsive promoters. Using the accumulated information, various biosensors have been developed [11]. The number of biosensors based on the molecule-responsive promoters is expected to increase further in the future. When it is known that a target molecule can be detected by an investigated molecule-responsive promoter, it is possible to reduce the time, labor, and cost of developing the PoC device to detect the target molecule. Thus, to develop a method to utilize the molecule-responsive promoters provides a chance for efficient development of PoC devices. Here, we freeze-dried a molecule-responsive protein expression system in microchambers of a microfluidic device. Furthermore, we employed two types of molecule-responsive promoters as proof-of-concept models: A quorum sensing molecule-responsive promoter [12,13] and a mercury-ion-responsive promoter [14,15]. The GFP-encoding genes under these two promoters were freeze-dried together with a CFPS system in the microchambers of the microfluidic device. Using the developed device, we demonstrated the target molecule sensing in the sample solutions as small as 1 µL.

## 2. Materials and Methods

### 2.1. Fabrication of the Microfluidic Device

**Figure 1:**
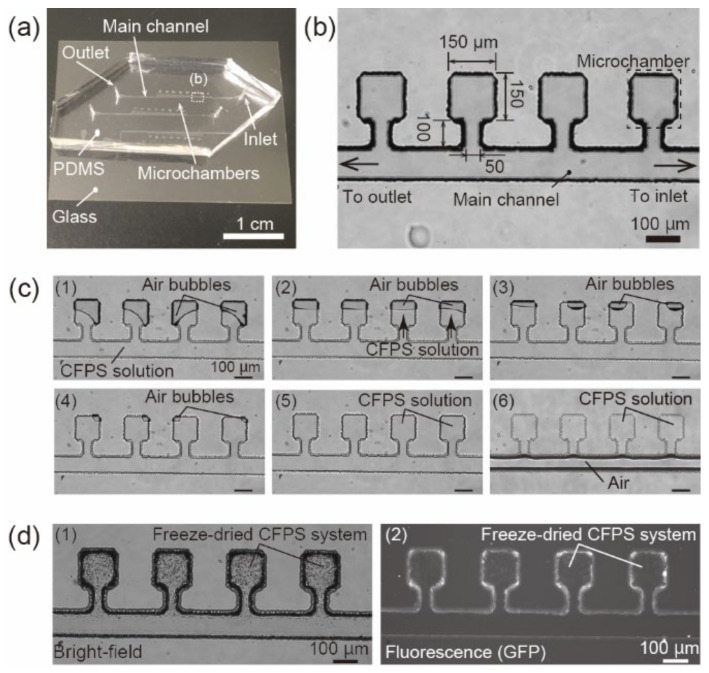
Fabrication procedures of the microfluidic device integrated with the cell-free protein synthesis (CFPS) system. (**a**) Image of the whole microfluidic device. (**b**) Microscopic image of the microchambers in the microfluidic device. (**c**) Time-lapse microscopic images during the procedures to introduce the CFPS solution in the microchambers. (**d**) Bright-field image (d1) and fluorescent image (d2) of the freeze-dried CFPS system in the microchambers. The freeze-drying condition was ~10 mTorr, −20 °C, 3 h.

The microfluidic device was fabricated using standard soft-lithography [16]. An SU8 mold (65 µm in height) was fabricated on a 2-inch Si wafer. Polydimethylsiloxane (PDMS) replica, shaping microchannels and microchambers, was fabricated using the SU8 mold. Inlets and outlets were fabricated by establishing through-holes in the PDMS replica with a biopsy punch of 0.5 mm in diameter. The PDMS replica was permanently bonded to cover glass (C030401 30 × 40 No. 1, Matsunami Glass, Osaka, Japan) via oxygen plasma bonding to complete the microfluidic device. The microfluidic device consisted of a main channel between the inlet and outlet and the microchambers (Figure 1a). The dimensions of the main channel and the microchambers were determined such that 1 µL of sample solutions filled the main channel and the microchambers. The width of the main channel was 100 µm and the height was 65 µm. The dimensions of the microchambers were 150 µm × 150 µm × 65 µm in width, depth, and height, respectively (Figure 1b). Microchambers were connected to the main channel through a narrow, 50 µm wide channel. The narrow part is required to prevent air penetration into the microchambers when isolation of the CFPS solution or sample solutions in the microchambers.

### 2.2. Construction of the Molecule-Responsive DNA

In this study, we constructed two types of molecule-responsive DNAs. These DNAs were activated to synthesize GFP in response to *N*-acyl-homoserine-lactone (AHL) or Hg^2+^. The procedures to construct these DNAs are described below.

AHL-responsive DNA was constructed from pRMC004, encoding the LuxI promoter, and pBEST-OR2-OR1-Pr-UTR1-deGFP-T500 encoding the green fluorescent protein (GFP). pRMC004 was a gift from Robert Cooper of the University of California, San Diego, CA, USA). pBEST-OR2-OR1-Pr-UTR1-deGFP-T500 (Addgene plasmid #40019; http://n2t.net/addgene:40019; RRID: Addgene_40019) was a gift from Vincent Noireaux of the University of Minnesota (Minneapolis, MN, USA) [17]. The LuxI promoter and the backbone containing ColE1 origin and Kanamycin resistance gene were subcloned using PCR from pRMC004. The deGFP gene was subcloned from pBEST-OR2-OR1-Pr-UTR1-deGFP-T500. These PCR products were purified and connected using NEBuilder HiFi DNA Assembly Master Mix (New England Biolabs, Ipswich, MA, USA). The constructed circular DNA was transformed into DH5α chemically competent cells (Takara, Kusatsu, Shiga, Japan). The plasmid DNA (pAHL-deGFP; Plasmid sequence S1) was amplified by culturing the cells and purified using the GenElute HP Plasmid Maxiprep Kit (Merck, Darmstadt, Germany). The pAHL-deGFP plasmid encodes LuxR from *Vibrio fischeri* under its native promoter and deGFP under the LuxI promoter. The LuxI promoter is activated by the AHL-luxR complex; therefore, deGFP is expressed in response to AHL.

Mercury-responsive DNA consisted of a mercury-responsible promoter (P_mer_) and deGFP gene. The P_mer_ part also encoded the MerR protein under a constitutive promoter. P_mer_ was activated by the MerR-Hg^2+^ complex and repressed by the MerR dimer [18]. The deGFP gene was located after P_mer_. Therefore, GFP expression occurs when Hg^2+^ ions are present; conversely, no GFP expression occurs when Hg^2+^ ions are not present. The DNA sequence of P_mer_ was obtained from the literature [19]. The DNA for the P_mer_ was synthesized by a DNA-synthesizing company (Genewiz, South Plainfield, NJ, USA). The DNA sequence, except for the P_mer_, was subcloned from pBEST-OR2-OR1-Pr-UTR1-deGFP-T500. These two DNAs were connected using NEBuilder HiFi DNA Assembly Master Mix. The constructed circular DNA was transformed into DH5α chemically-competent cells (Takara). The plasmid DNA (pHg-deGFP; Plasmid sequence S2) was amplified by culturing the cells and purified using the GenElute HP Plasmid Maxiprep Kit.

These DNAs were further concentrated using the QIAprep Spin Miniprep Kit (Qiagen, Hilden, Germany) when necessary.

### 2.3. Preparation of the Molecule-Sensing Cell-Free Protein Synthesis System

The CFPS system was purchased from Promega (S30-T7-High-Yield-Protein-Expression-System; Madison, WI, USA). We used the CFPS system according the manufacturer’s instructions. To sense AHL, pAHL-deGFP was added to the CFPS system; similarly, to sense mercury ions, pHg-deGFP was added.

### 2.4. Freeze-Drying of Cell-Free Protein Expression System and DNA in the Microfluidic Device

The CFPS solution, containing DNA (pBEST-OR2-OR1-Pr-UTR1-deGFP-T500, pAHL-deGFP, or pHg-deGFP in this study), was introduced into the microchambers of the microfluidic device and freeze-dried. First, the microfluidic device was placed in a vacuum chamber for 20 min. Next, 1 µL of the CFPS system, containing the designated concentration of DNA, was injected from the inlet using a pipette. At this time, the CFPS solution was absorbed into the microchambers (Figure 1c (1)–(5)). The air bubbles in the microchambers were removed via out-diffusion through the PDMS wall because PDMS is a gas-permeable material [20]. After the microchambers were completely filled with the CFPS solution, air was injected from the inlet using a pipette. The typical procedure was to constantly inject 200 µL of air in approximately 0.8 s. The injection rate in this case was calculated to be 15 mL/min. From 7.5 to 30 mL/min can be used for this air injection procedure (Figure S1). After air injection, the CFPS solution was confined in the microchambers (Figure 1c (6)). The microfluidic device was pre-frozen at −80 °C for 30 min. The device was then freeze-dried at ~10 mTorr at −20 °C for 3 h using a freeze-dryer (Advantage EL 85; SP Industries, Warminster, PA, USA) with a vacuum pump (GLD-051; Ulvac, Kanagawa, Japan) or ~30 mTorr at −80 °C for 6 h using a freeze-dryer (VD-500F, Taitec, Aichi, Japan) with another vacuum pump (GCD-051X, Ulvac). The used freeze-drying condition was noted along with descriptions of respective experiment. After freeze-drying, the CFPS system appeared as dried particles in the microchambers (Figure 1d (1)). The freeze-dried CFPS system showed slight self-fluorescence when observed using a fluorescent microscope (Ti2-E; Nikon, Tokyo, Japan) with a GFP filter unit (Figure 1d (2)). The microfluidic device was kept at room temperature until further use.

### 2.5. Rehydration to Test the Aqueous Sample

**Figure 2:**
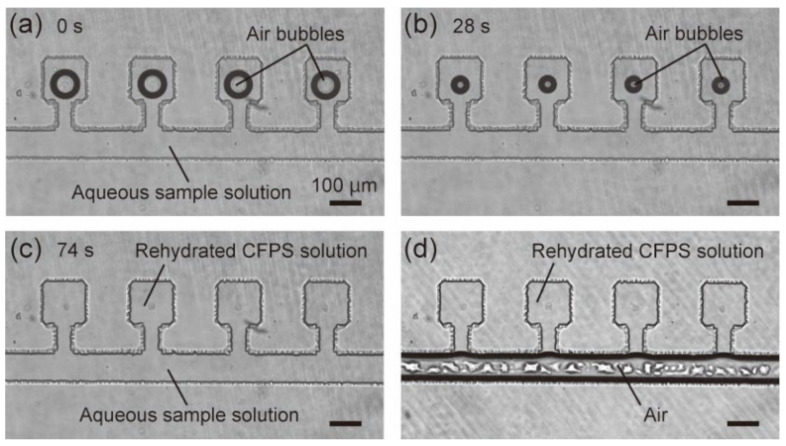
Rehydration of freeze-dried CFPS system in microchambers using an aqueous sample solution. (**a**–**c**) Microscopic time-lapse images of the microchambers after injecting 1 μL of the aqueous sample solution from the inlet. (**d**) Microscopic images of the microchambers filled with the rehydrated CFPS solution. The freeze-drying condition was ~10 mTorr, −20 °C, 3 h.

To test the aqueous sample, the microfluidic device was first placed in the vacuum chamber for 20 min. Next, 1 µL of the sample solution was injected from the inlet using a pipette. After injection, the sample solution was absorbed into the microchambers to rehydrate the freeze-dried CFPS system. The remaining air bubbles in the microchambers were absorbed into the PDMS walls to disappear typically within 2 min (Figure 2a–c). Finally, the air was injected from the inlet 2 min after starting rehydration to confine the rehydrated CFPS solution in the microchambers (Figure 2d). The air injection procedure should be done as soon as possible after rehydration to avoid dilution of the components of the CFPS system (Figure S2).

### 2.6. Fluorescent Imaging of Cell-Free Protein Synthesis Solution

Fluorescent intensities of the CFPS solutions were monitored using a fluorescent microplate reader (Infinite F200; Tecan, Zürich, Switzerland) with a fluorescent filter unit for GFP. 9.5 µL of the sample solutions were applied to each well of a 384-well plate (Merck). During the measurement, the microplate was kept at 37 °C so that the CFPS system synthesized proteins at high speed. The obtained fluorescence intensities (F) were normalized by the initial intensities (F_0_) as (F−F_0_)/F_0_ (denoted as ΔF/F_0_).

### 2.7. Fluorescence Imaging of Microchambers

Fluorescence intensities of the microchambers were monitored using an inverted fluorescent microscope (Ti2-E; Nikon) with a fluorescent filter unit for GFP (GFP HQ; Nikon) and a monochromic CCD camera (CoolSNAP HQ2; Teledyne Technologies, Thousand Oaks, CA, USA). Before monitoring, the inlet and outlet were sealed with vacuum grease to prevent dehydration. The microfluidic device was then placed in a Petri dish with water droplets to maintain the humidity. The microfluidic device in the Petri dish was maintained at 37 °C using a heating plate (PTi-108RX; Tokai Hit, Shizuoka, Japan). The obtained fluorescent images were analyzed using image processing software (ImageJ; NIH, Bethesda, MD, USA). The fluorescence intensities in the microchambers (F) were normalized by the initial intensities (F_0_) as (F−F_0_)/F_0_ (denoted as ΔF/F_0_).

## 3. Results and Discussion

### 3.1. Cell-Free Protein Synthesis from Freeze-Dried DNA

**Figure 3:**
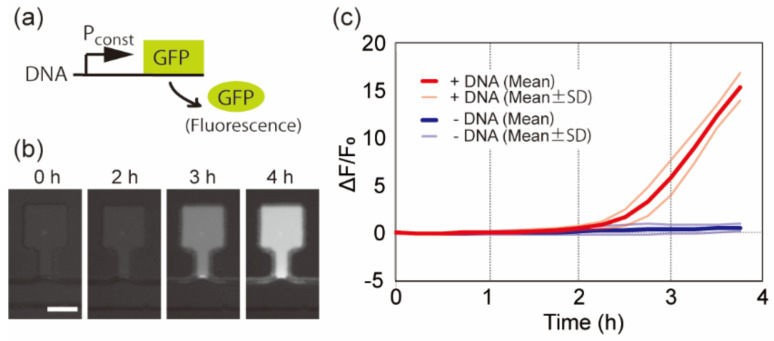
Protein synthesis using freeze-dried CFPS system and freeze-dried DNA. (**a**) Schematic diagram of the gene circuit demonstrated in (**b**,**c**). (**b**) Time-lapse fluorescent images of the representative microchamber freeze-dried together with the DNA shown in (**a**). The scale bar is 100 µm. (**c**) Time-course of the fluorescent intensities in the microchambers with (+DNA) or without DNA (−DNA). SD shows standard deviation (*n* ≧ 3 each). The freeze-drying condition of the microfluidic device was ~30 mTorr, −80 °C, 6 h.

We first investigated the functionality of the freeze-dried CFPS system and the freeze-dried DNA. To do this, we freeze-dried the CFPS system together with 21 ng/µL of deGFP-encoding-DNA (pBEST-OR2-OR1-Pr-UTR1-deGFP-T500). The DNA encoded deGFP under a constitutive promoter (P_const_) (Figure 3a). We rehydrated them using an aqueous buffer solution containing 5 mM Tris-HCl (pH 8.0) and 0.5 mM ethylenediaminetetraacetic acid (EDTA). After rehydration, the fluorescence intensities in the microchambers gradually increased, especially after 2 h, as shown in Figure 3b. The increase in fluorescence intensity was caused by GFP expression, since the distributions of the fluorescent intensities between the samples with and without DNA were separated after 3 h (Figure 3c). This result shows that the freeze-dried CFPS system and the freeze-dried DNAs remain functional for gene expression after rehydration.

### 3.2. AHL Sensing

**Figure 4:**
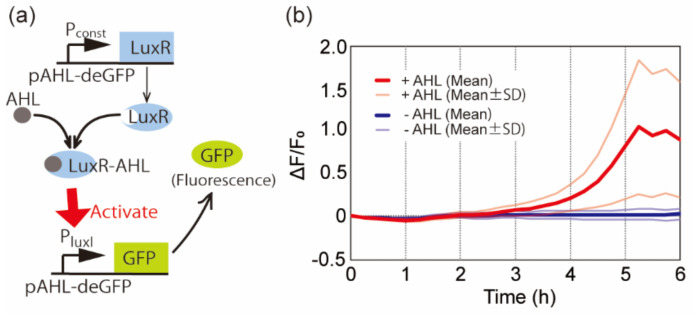
(**a**) Schematic diagram of *N*-acyl-homoserine-lactone (AHL) sensing using AHL-responsive DNA (pAHL-deGFP). (**b**) Time-course of the fluorescence intensities in the microchambers with the gene circuit shown in (a). The time interval between each image acquisition was 15 min. SD shows standard deviation (*n* ≧ 3 each). The freeze-drying condition of the microfluidic device was ~30 mTorr, −80 °C, 6 h.

We next examined biomolecule sensing. The target biomolecule was AHL, which is widely used in bacterial quorum sensing [21]; therefore, AHL is an indicator of the existence of bacteria. We freeze-dried the CFPS solution containing 84 ng/µL of pAHL-deGFP. pAHL-deGFP encoded the LuxR gene under a constitutive promoter (P_const_) and deGFP gene under the LuxI promoter (P_luxI_) (Figure 4a). The AHL-sensing DNA circuit worked intricately. First, the LuxR protein was expressed under P_const_. When the concentration of AHL was high enough, the LuxR molecule bound to AHL to form the LuxR-AHL complex [12]. The LuxR-AHL complex then bounds to the P_luxI_ promoter to activate gene expression under this promoter. As a result, the DNA circuit expressed deGFP in response to AHL concentrations higher than ~20 nM. The aqueous solution contained 5 mM Tris-HCl (pH 8.0) and 0.5 mM EDTA with or without 10 μM AHL. After rehydration with 1 μL of the aqueous solution containing AHL, the average fluorescence intensity from the microchambers gradually increased (Figure 4b). Meanwhile, rehydration without AHL did not cause an increase in the average fluorescent intensity from the microchambers. This result indicates that the microfluidic device freeze-dried with the CFPS system and pAHL-deGFP detects the AHL molecule.

### 3.3. Mercury Sensing

#### 3.3.1. Characterization of Mercury Sensing DNA

We finally examined mercury ion sensing. Drinking water and sources of drinking water (usually rivers) are supposed to be quality checked periodically. The inspection items include contaminants that can be toxic to human health, such as mercury, cadmium, arsenic, lead, chromium, polychlorinated biphenyl, cyanide, and benzene [22,23]. Among them, mercury is highly toxic to the human body. Therefore, we applied the developed microfluidic device to detect mercury ions in water samples.

**Figure 5:**
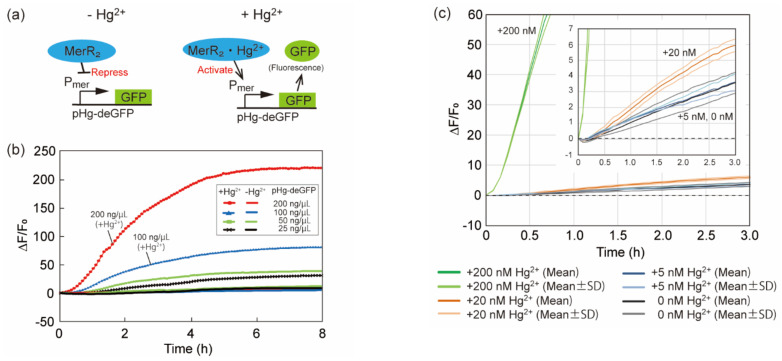
Characterization of mercury sensing DNA (pHg-deGFP). (**a**) Schematic diagram of mercury sensing using the mercury sensing DNA. (**b**) Time-course of the fluorescent intensities of the CFPS solution with various pHg-deGFP concentrations. +Hg^2+^ and −Hg^2+^ represent the sample with/without 200 nM of Hg^2+^ respectively. (**c**) Time-course of the average fluorescent intensities of the CFPS solution with various Hg^2+^ concentrations. The CFPS solution contained 200 ng/μL of pHg-deGFP. The inset shows the magnified graph around ΔF/F_0_ = 0. Curves with the dark colors represent average (Mean). Curves with the light colors represent average ± standard deviation (*n* = 3) (Mean ± SD). The time interval between each data acquisition in (**b**,**c**) was 5 min.

First, we confirmed that the constructed mercury sensing DNA (pHg-deGFP) could detect Hg^2+^ ions. pHg-deGFP was expected to synthesize GFP when the Hg^2+^ concentration was higher than the threshold, as shown in Figure 5a.

Then, we investigated which pHg-deGFP concentration was suitable for the detection of Hg^2+^ ions. Figure 5b shows GFP expression levels with or without 200 nM of Hg^2+^ at various concentrations of pHg-deGFP. This indicates that as the pHg-deGFP concentration increased, GFP expression levels also increased in the presence of 200 nM of Hg^2+^ ions. It also shows that even if the pHg-deGFP concentration was high, GFP expression levels did not change dramatically in the absence of Hg^2+^ ions. Therefore, higher concentrations (≥ 100 ng/µL) of pHg-deGFP are suitable for the detection of Hg^2+^ ions, in fact 200 ng/µL was used for subsequent experiments.

Next, we investigated the detection limit of the mercury sensing system. Figure 5c shows the GFP expression level at various concentrations of Hg^2+^. The graph shows that 20 nM of Hg^2+^ was detected without overlapping error bars against 0 nM. However, 5 nM of Hg^2+^ was not detectable because the error bars of 5 and 0 nM overlapped. From this result, the detection limit was found to be 20 nM. This value is lower than the WHO standard (~30 nM) [24].

#### 3.3.2. Detection of Mercury Ions in the Microfluidic Device

**Figure 6:**
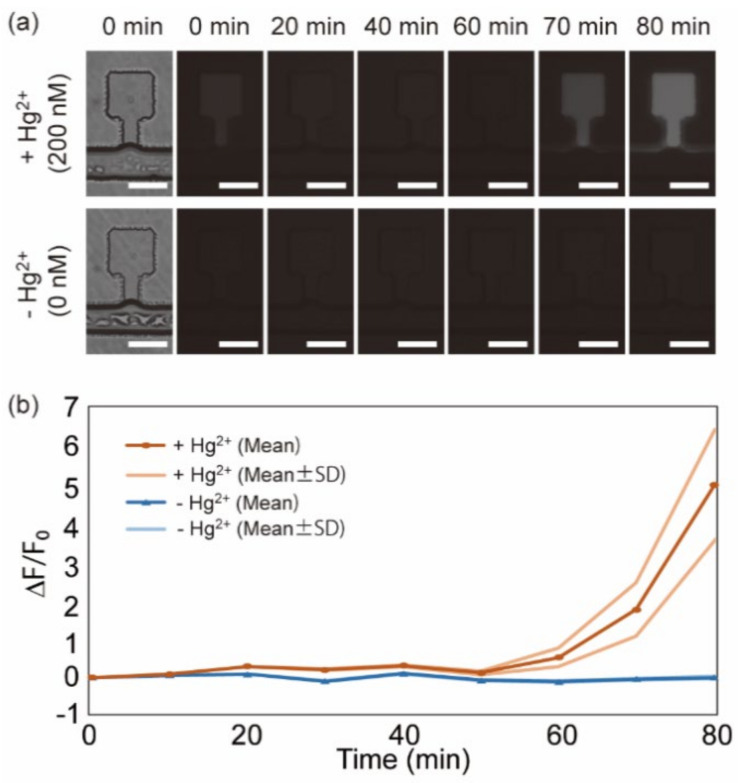
Detection of mercury ions in the water sample. (**a**) Microscopic images of the representative microchambers with 200 nM of Hg^2+^ (upper row) and 0 nM of Hg^2+^ (lower row). Bright-field images at the initial state (left end) and time-lapse fluorescence images from 0 to 80 min. The exposure time was 5 s. The time interval between acquiring images was 10 min. The scale bars are 100 µm. (**b**) Time-course of the average relative fluorescence intensities in the microchambers with water containing 200 nM of Hg^2+^ (+Hg^2+^) or 0 nM of Hg^2+^ (−Hg^2+^). 200 ng/µL of pHg-deGFP was contained in the CFPS solution when freeze-drying. Curves with the dark colors represent average values (Mean). Curves with the light colors represent average ± standard deviation (*n* = 4) (Mean ± SD). The freeze-drying condition of the microfluidic device was ~10 mTorr, −20 °C, 3 h.

We next confirmed that mercury ions could be detected in the developed microfluidic device with the freeze-dried CFPS system and the freeze-dried pHg-deGFP DNA. We freeze-dried the CFPS solution containing 200 ng/µL of pHg-deGFP in the microchambers of the microfluidic device. After freeze-drying, the freeze-dried CFPS system with pHg-deGFP in the microchambers was rehydrated with water with/without 200 nM Hg^2+^ (Milli-Q water with/without 200 nM of HgCl_2_). Figure 6a shows the fluorescent time-lapse images of the microchambers after rehydration. Figure 6b shows the time-course of average relative fluorescent intensities in each condition. These indicate that the fluorescence intensities only increase in the microchambers with 200 nM of Hg^2+^. From this result, we conclude that the developed microfluidic mercury sensor could detect Hg^2+^ ions.

#### 3.3.3. Testing the Microfluidic Devices with Water Samples from the River

**Figure 7:**
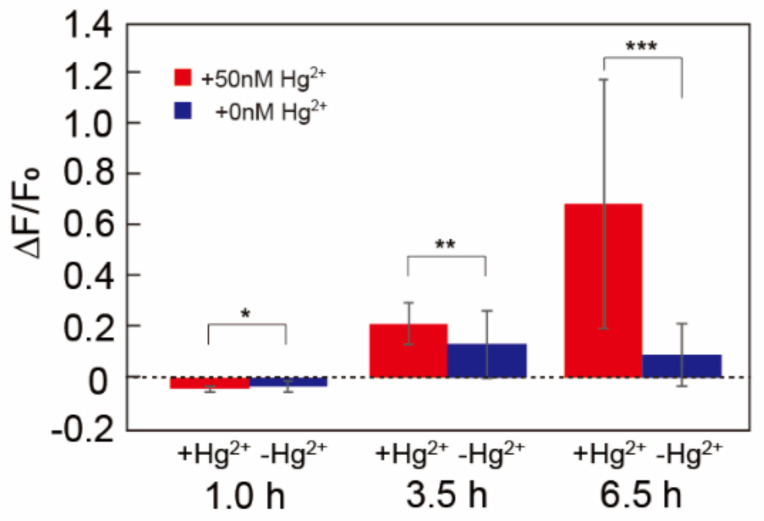
Detection of mercury ions in a water sample from the Kamo river. Average relative fluorescence intensities in the microchambers with river water containing 50 nM of Hg^2+^ (+50 nM Hg^2+^, red bars) and non-treated river water (+0 nM Hg^2+^, blue bars) after 1.0, 3.5, and 6.5 h. The river water sample with 50 nM of Hg^2+^ was prepared by dissolving HgCl_2_ into the river water. The water samples were injected in the microfluidic device at 0 h. Error bars represent standard deviation (*n* = 4). * *p* < 0.5, ** *p* < 0.5, *** *p* < 0.05 (Welch’s *t*-test) [25]. The freeze-drying condition of the microfluidic device was ~10 mTorr, −20 °C, 3 h.

We demonstrated that water samples obtained from a river could be handled/detected with the developed microfluidic mercury sensor. Water samples were obtained from the Kamo River in Kyoto. We dissolved HgCl_2_ to obtain 50 nM of Hg^2+^ in one of the water samples. The water samples were directly applied to the microfluidic device without any treatment. Figure 7 shows the average relative fluorescence intensities in the microchambers at each time point in each sample. After 6.5 h, the *p*-value was calculated to be ~0.04 using Welch’s *t*-test. This result indicates that 50 nM of Hg^2+^ in the river water sample is detected after 6.5 h. From this result, we conclude that the developed device can be applied for water quality testing/monitoring. The variance of fluorescent intensities in Figure 7 is relatively large. This is possibly because Hg^2+^ concentration in this sample solution (50 nM) is close to the dissociation constant between MerR and Hg^2+^ (93 nM [18]). Around the concentration of the dissociation constant, binding probability of MerR-Hg^2+^ complex to pHg-deGFP DNA should dramatically change. Therefore, small differences of Hg^2+^ concentrations between the microchambers may result in relatively large variance of the fluorescent intensities.

## 4. Conclusions

This study aimed to develop a biosensing device that can be used via simple operation with a small volume of samples. To achieve this concept, we developed a microfluidic device that has a freeze-dried CFPS system in its microchambers. The device was fabricated by freeze-drying the CFPS solution in the microchambers. To prove this concept, we first validated that the freeze-dried CFPS system can express proteins from freeze-dried DNA after rehydration with 1 µL of the aqueous solution injected from the inlet. Next, we demonstrated two types of biosensing, AHL and Hg^2+^ sensing, using the developed device integrated with the freeze-dried CFPS system. For sensing AHL and Hg^2+^, the AHL-responsive promoter and the Hg^2+^-responsive promoter were employed. By having these promoters before the GFP gene in the DNA sample, DNA activation by AHL or Hg^2+^ was enabled and could be validated through the GFP signal. Using the developed device with these DNA samples, we detected 10 µM of AHL and 200 nM of Hg^2+^. Notably, the simple operation via the injection of an aqueous sample solution as low as 1 µL enables biosensing. We also demonstrated water quality testing with this microfluidic device by injecting 1 µL of sample obtained from the river into the device: We could detect 50 nM of Hg^2+^ that was artificially added to the river water.

As shown in this study, the microfluidic device developed with the freeze-dried CFPS system enables small-volume biosensing. In addition, by changing the promoter region in the DNA, sensing devices responsive to other molecules can be developed. Therefore, this technology will be useful for on-site biosensing applications such as PoC diagnostics and water quality testing.

## Supplementary Materials

The following are available online at https://www.mdpi.com/2072-666X/12/1/27/s1, Plasmid sequence S1: pAHL-deGFP, Plasmid sequence S2: pHg-deGFP, Figure S1: Isolation of the microchambers by air injection at various flow rates, Figure S2: Averaged relative fluorescence intensities (ΔF/F_0_) of the microchambers at 80 min with various time period between rehydration and isolation of the microchambers.
