# Supplementary material for: Microfluidic Device with an Integrated Freeze-Dried Cell-Free Protein Synthesis System for Small-Volume Biosensing
Source: Micromachines (Basel). 2020 Dec 29;12(1):27. doi: 10.3390/mi12010027 (PMC7824204; doi:10.3390/mi12010027)
Supplement: Supplementary file 1 [file micromachines-12-00027-s001.zip › micromachines-1039212-supplementary.pdf]

micromachines

MDPI

Supplementary Materials: Microfluidic Device with an Integrated Freeze-Dried Cell-Free Protein Synthesis System for Small-Volume Biosensing

Taishi Tonooka

1. Validation of the Flow Rate of Air Injection to Isolate the Microchambers

To isolate the CFPS solution or the sample solution in the microchambers, air was injected from the inlet. The typical procedure for air injection was constant injection of 200 μL of air in about 0.8 s using a pipette. The average flow rate in this case is calculated to be 15 mL/min. The flow rate may be user-dependent. Therefore, the acceptable flow rate was investigated. Figure S1 shows microscopic images of the microchambers before/after air injection at designated flow rate. From this result, flow rate between 7.5 mL/min to 30 mL/min is acceptable to isolate the microchambers.

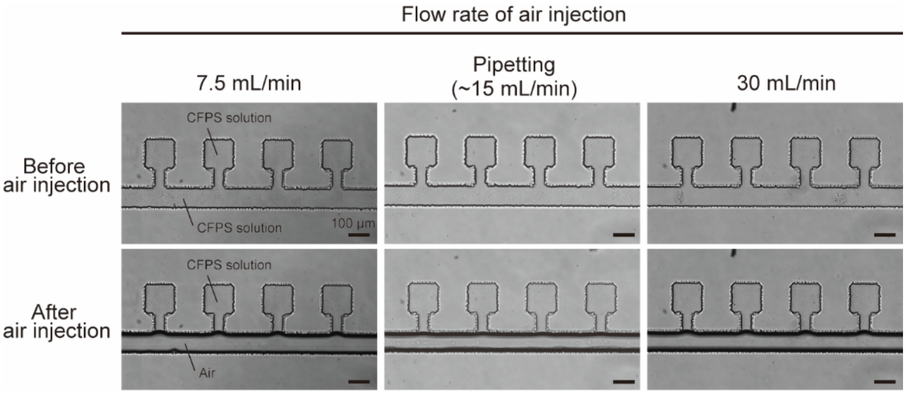

Figure 1. Isolation of the microchambers by air injection at various flow rates. Flow rates of 7.5 and 30 mL/min were regulated by a syringe pump. Pipetting shows the typical procedure of air injection (200 μL in 0.8 s), which corresponds to 15 mL/min.

2. Dependency on Time Period until Confinement of the Sample Solution in the Microchambers during Rehydration Process

When testing the sample solution, the freeze-dried CFPS system in the microchambers are rehydrated. After rehydration, air is injected to isolate the microchambers. Air injection was performed at 2 min after starting rehydration because rehydration process typically finished within 2 min to fill the sample solution in the microchambers. During this process, the components of CFPS system can diffused out of the microchambers. Therefore, the influence of time period until confinement of the sample solution in the microchambers on generated fluorescent signal was investigated.

To investigate it, the CFPS solution containing 200 ng/μL of pHg-deGFP was freeze-dried in the microchambers. The freeze-drying condition was ~10 mTorr, −20°C, 3 h. At the rehydration step, 1 μL of 2 μM $HgCl_2$ aqueous solution was injected into the device (Time = 0 min). Air was injected 2 min, 3 min, and 5 min after starting rehydration in each device. Figure S2 shows average $\Delta F/F_0$ at 80 min in each condition. The graph indicates that, as time period after rehydration becomes longer, the averaged relative fluorescence intensity becomes smaller. This result shows that the long time period after rehydration significantly reduce the activity of the rehydrated CFPS system probably due to out-diffusion of the components of the CFPS system. It is important to fix the time period between rehydration and isolation of the microchambers to obtain consistent results.

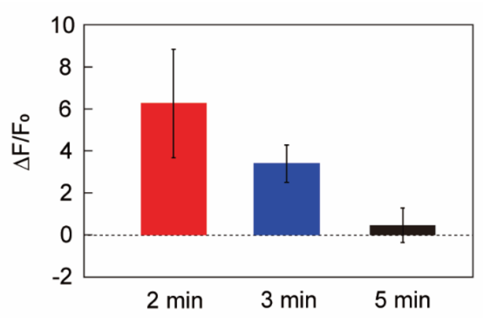

Figure 2. Averaged relative fluorescence intensities ($\Delta F/F_0$) of the microchambers at 80 min with various time period between rehydration and isolation of the microchambers. 2 min, 3 min, and 5 min represent time period between injection of the sample solution and injection of air. Error bars represent standard deviation (n = 4).
